# Supplementary material for: Tumor Site-Specific Cleavage Improves the Antitumor Efficacy of Antibody–Drug Conjugates
Source: Int J Mol Sci. 2023 Jul 2;24(13):11011. doi: 10.3390/ijms241311011 (PMC10342105; doi:10.3390/ijms241311011)
Supplement: Supplementary file 1 [file ijms-24-11011-s001.zip › ijms-2454571-supplementary.pdf]

Supplementary Materials

1. Methods of the supplementary experiments

1.1. Linker cleavage by uPA

Fc-U-Z_{HER2} (20 μM) was incubated in the presence of 400 nM uPA (Sino Biological, Beijing) at 37 °C for 1, 2, 4, 6, 10 and 24 h. To stop the reaction, 20 μL of sample was incubated with 5 μL of protein reducing buffer (GeneStar, Beijing) containing 1 mM DTT for 5 minutes at 95 °C. Cleavage was confirmed by 12.5% SDS–PAGE and staining with Coomassie Blue R250.

1.2. Expression and purification of sortase A

The sequence encoding Ca^{2+}-dependent sortase A from *Staphylococcus aureus*, with a glutathione S-transferase (GST) tag added to the C-terminus for purification, was inserted between the Nco I and Xho I restriction sites of the vector pET-28a (+) and transformed into BL21（DE3）pLys competent cells. For expression, bacteria in medium containing ampicillin were induced with 0.5 mM isopropyl β-D-1-thiogalactopyranoside (IPTG) overnight at 220 rpm and 16 °C. After harvesting by centrifugation (4000 rpm, 20 min, 4 °C), the pellet was disrupted with a high-pressure homogenizer and purified by a GST affinity chromatography column to afford sortase A. The purity of the protein was analyzed by 12.5% SDS–PAGE.

1.3. Exploration of the reaction conditions for sortase A-mediated conjugation

The coupling reaction conditions catalyzed by sortase A were explored including the influence of GGG-VC-PAB-MMAE equivalence, sortase A concentration, reaction time and reaction temperature on the reaction efficiency. The reactions were carried out by mixing Fc-U-Z_{HER2} protein (40 μM), GGG-VC-PAB-MMAE (0.08 – 1.6 mM, 2 – 40 equiv.) and sortase A (0.1 – 5.0 μM) in sortase buffer (50 mM Tris-HCl, 150 mM NaCl and 10 mM $CaCl_2$ at pH 7.4) and shaking at 4 °C, 25 °C or 37 °C for 1 to 24 h. To analyze the reaction efficiency, 20 μL of the samples were taken and incubated with 5 μL of protein reducing buffer (GeneStar, Beijing) containing 1 mM DTT for 5 min at 95 °C, followed by analysis with 12.5% SDS–PAGE.

1.4. Preliminary stability test of Fc-U-Z_{HER2}-MMAE by SDS–PAGE.

PBS solution with Fc-U-Z_{HER2}-MMAE (1 mg/mL) was stored at -80 °C and -20 °C for 3 and 7 days and then analyzed by SDS–PAGE with Fc-U-Z_{HER2} as the control.

1.5. Binding selectivity verified by flow cytometry and microscopy

NCI-N87, BT-474, SK-BR-3, SK-OV-3, and H-CC70 cells were seeded at a density of 1×10^5 cells per well in 12-well plates overnight, and then 10 nM conjugate or primitive protein was added. After incubation for 1 hour, a mouse anti-MMAE monoclonal antibody was added for 1 hour of incubation, followed by 1 hour of incubation with the FITC-labeled goat anti-mouse antibody on ice. The cells were collected and then analyzed by flow cytometry using a FACSCalibur instrument (BD Biosciences, San Jose, CA, USA). CellQuest Pro (BD Biosciences, San Jose, CA, USA) software was used for data acquisition, and FlowJo 10 (Tree Star Inc., Ashland, OR, USA) was used for result analysis.

For the cellular receptor binding study, NCI-N87 cells highly expressing HER2 and H-CC70 cells with low HER2 expression were plated on chamber glass slides at a density of 1×10^5 cells/well. The conjugates and the primitive proteins were added to the culture medium at a concentration of 100 nM and incubated for 2 h. After fixing with a paraformaldehyde solution, a mouse anti-MMAE monoclonal antibody or a mouse anti-His monoclonal antibody was added to the wells as the primary antibody (1 μg/mL) and incubated for an additional 1 h. FITC-labeled goat anti-mouse antibody diluted in PBS (1:5000) was added as the secondary antibody for 1 h of incubation. The nuclei were stained with 4'-6-diamidino-2-phenylindole (DAPI). Cells were rinsed and visualized by CLSM. Each staining experiment was performed on three individual groups, and the photographs in the figures are representative of each group.

1.6. Subcellular colocalization assays

High HER2-expressing NCI-N87 cells and low HER2-expressing H-CC70 cells were seeded at approximately 1×10^5 cells/dish in 35 mm confocal dishes for 24 h. Then, fluorescein isothiocyanate (FITC)-labeled conjugates or controls were added to the culture medium at a concentration of 100 nM and incubated at 37 °C for 4 h. Here, the conjugates and the control proteins were prelabeled with FITC by incubating the proteins with an 8-fold molar excess of FITC (US Everbright Inc., Jiangsu, China) in 0.1 M $NaHCO_3$ buffer for 5 h at room temperature in the dark, and then the excess FITC was removed with a PD-10 desalting column (GE Healthcare, Boston, MA, USA). The cells were washed three times with PBS, incubated with Hoechst 33342 (blue fluorescence) to stain the nuclei, and further treated with 50 nM LysoTracker Red DND-99 for 30 min at 37 °C to stain the lysosomes. Confocal images of the stained live cells were captured using confocal laser scanning microscopy (CLSM; TCS SP5, Leica, Hessian, Germany).

1.7. Visual observations of conjugates in vitro cytotoxicity through live/dead staining

SK-BR-3 cells with high HER2 expression and H-CC70 cells with low HER2 expression were seeded in 96-well plates at a cell density of 5000 cells/well and cultured at 37 °C for 24 h. Then, 100 nM, 1 nM, and 10 pM conjugates were added to the wells of SK-BR-3 cells, or 100 nM conjugates were added to the wells of H-CC70 cells, and incubated for another 72 h at 37 °C. After washing with PBS, the cells were stained with calcein-AM and ethidium homodimer-1 (EthD-1) and then imaged under an inverted microscope (DMI 4000B, Leica, Hessian, Germany).

1.8. Flow cytometry analysis of cell apoptosis

An Annexin V-APC/PI apoptosis detection kit (Beyotime Biotech Inc., Shanghai, China) was used according to the manufacturer's instructions. Here, SK-BR-3 cells and H-CC70 cells were seeded at a density of 2×10^5 overnight and treated with 10 nM conjugates for 24, 48, or 72 h. After digestion with trypsin without EDTA, all the cells were collected by centrifugation at 300 × g for 5 min and then washed with cold PBS twice. Annexin V-APC and PI staining solutions were added for 20 min of incubation. Then, all samples were analyzed by flow cytometry using a FACSCalibur instrument (BD Biosciences, San Jose, CA, USA). The software CellQuest Pro (BD Biosciences, San Jose, CA, USA) and FlowJo 10 (Tree Star Inc., Ashland, OR, USA) were used for data acquisition and analysis of the results, respectively.

1.9. Selective induction of G2 growth arrest via flow cytometry analysis

NCI-N87 cells and H-CC70 cells were seeded in a 6-well plate at a density of 5×10^5 cells per well and incubated for 24 h before being replaced with medium containing a final concentration of 100 nM conjugates; blank medium was used as the control. After incubation for 18 h, the cells were collected into a single-cell suspension with precooled PBS. Then, the cells were fixed by adding precooled absolute ethanol slowly to a final concentration of 75% and maintaining the temperature at -20 °C overnight (18 – 24 h). After washing the cells with precooled PBS twice, RNase (50 μg/mL) and PI (50 μg/mL) were added for another 30 min in the dark. Then, the cells were collected and analyzed via flow cytometry (BD FACS Calibur, BD Biosciences, San Jose, CA, USA), and ModFit software was used for data analysis.

1.10. Detection of uPA in mice by ELISA and immunohistochemistry

The relative concentration of the uPA enzyme in organs was analyzed in a subcutaneous

xenograft model of NCI-N87 tumors with high HER2 expression levels. Briefly, 5×10^6 cells were subcutaneously inoculated into the flank region of BALB/c nude mice. After the tumor volumes reached 100 – 200 mm^3, the mice were euthanized, and their organs (liver, heart, spleen, lung, kidney) and tumor tissues were dissected and homogenized. The homogenizing liquid was transferred to a centrifuge tube, and the supernatant was obtained by centrifugation. Both samples and standards were tested using the human uPA ELISA Kit (Cohesion Biosciences). The ELISA plates were measured with a microplate reader at 450 nm, and the content of uPA enzyme in each organ was calculated from the standard curve and organ weight. For immunohistochemistry, the mice were euthanized, and the tumors and normal organs, including heart, liver, spleen, lung, and kidney tissues, were excised to prepare paraffin sections for anti-uPA antibody (T56732, Ab-mart, Beijing, China) staining. Images were captured under a microscope (Nikon, Eclipse Ci-L, Tokyo, Japan) and analyzed by CaseViewer 2.4.

2. Results of the supplementary experiments

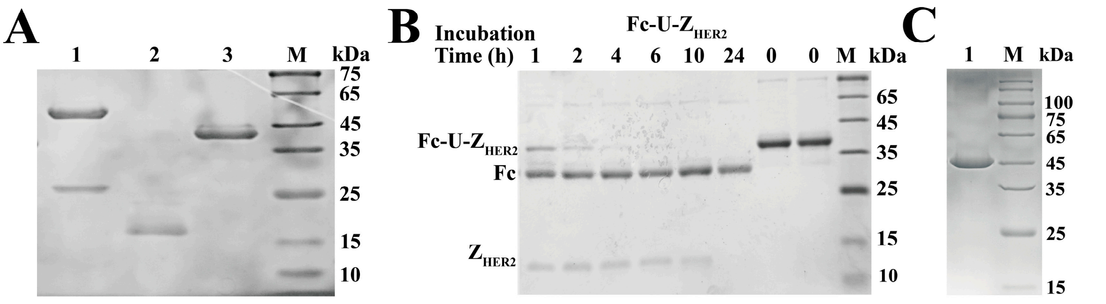

Fig. S1. SDS–PAGE and uPA cleavage of Fc-U-Z$_{HER2}$ and the other designed proteins. (**A**) Purified Herceptin (Lane 1), Z$_{HER2}$ (Lane 2), and Fc-U-Z$_{HER2}$ (Lane 3) were loaded onto a 12.5% polyacrylamide gel with protein weight standards and stained with Coomassie brilliant blue. (**B**) Fc-U-Z$_{HER2}$ (20 μM) was incubated in the presence of 400 nM uPA in buffer (50 mM Tris-HCl, 150 mM NaCl, 0.05% Tween-20, pH=7.4) at 37 °C. After 1, 2, 4, 6, 10 h and 24 h, 5 μL samples were taken, and 5×SDS–PAGE protein sample buffer (GeneStar) containing 20 mM 2-mercaptoethanol was added, followed by incubation for 5 min at 95 °C and detection with 12.5% SDS–PAGE. (**C**) Purified sortase A (Lane 1) with a GST tag detected by 12.5% SDS–PAGE.

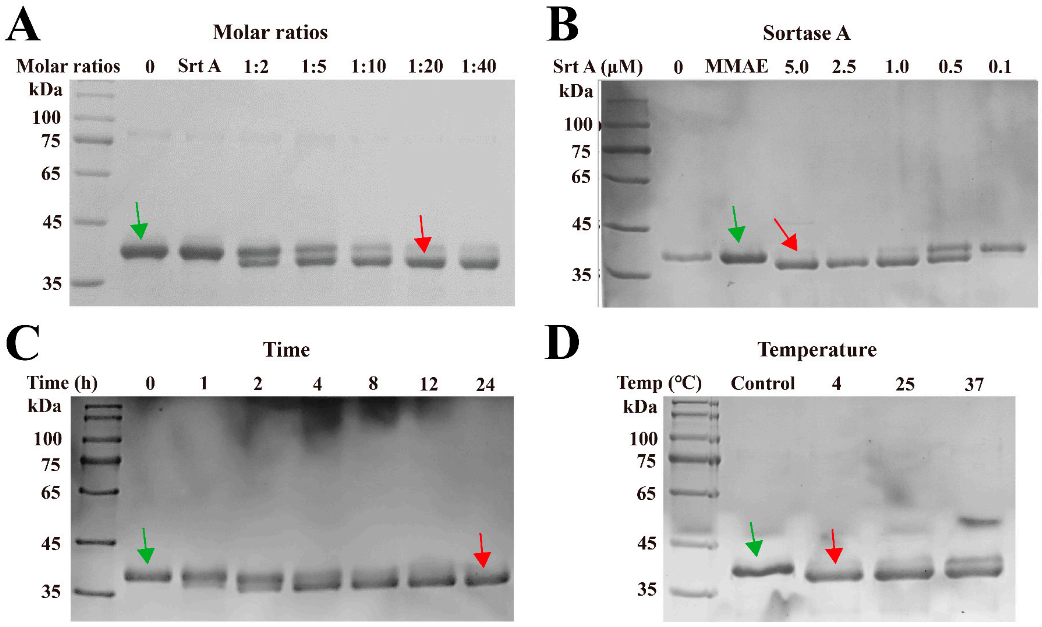

Fig. S2. Exploration of the reaction conditions for sortase A-mediated conjugation. (**A–D**) SDS–PAGE of the reactions with different parameters, including molar ratios (1:2 – 1:40) of Fc-U-Z$_{HER2}$ (40 μM) to GGG-VC-PAB-MMAE (**A**), concentrations of sortase A (0.1 μM – 5 μM) (**B**), reaction times (1 h – 24 h) (**C**) and reaction temperatures (4 °C, 25 °C and 37 °C) (**D**). Green arrows represent the raw proteins, and red arrows represent the products. Lane 0 and

control: Fc-U-ZHER2; Srt A: mixture of Fc-U-ZHER2 and sortase A; MMAE: mixture of Fc-U-ZHER2 and GGG-VC-PAB-MMAE.

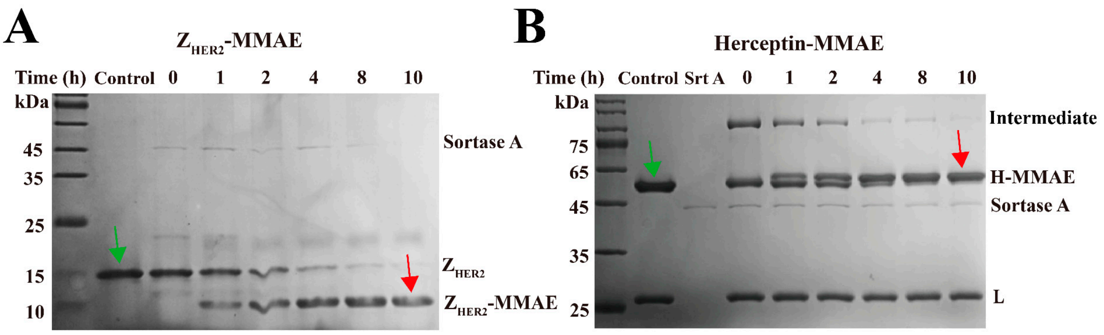

Fig. S3. Influence of reaction time on the efficiency of the sortase A-catalyzed coupling reaction involving other control proteins. (**A–B**) SDS–PAGE of the reactions mixing ZHER2 (**A**) or Herceptin (**B**) with 10-fold equivalents of GGG-VC-PAB-MMAE and 2.5 μM sortase A in 1× sortase buffer (final concentration). The reaction mixture was incubated at 4 °C for various times and then examined by SDS–PAGE as previously described. Green arrows represent the substrate proteins, and red arrows represent the products. Intermediate refers to the acyl-enzyme intermediate formed in the coupling reaction from the active site cysteine residue of soratse A and the threonine residue of the LPXTG tag.

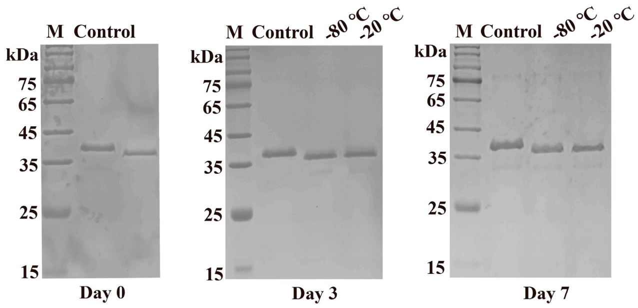

Fig. S4. Preliminary test of the stability of Fc-U-ZHER2-MMAE. A PBS solution of Fc-U-ZHER2-MMAE (1.0 mg/mL) was stored at -80 °C and -20 °C for 3 and 7 days and then analyzed by SDS–PAGE. The control lane refers to Fc-U-ZHER2 protein without MMAE.

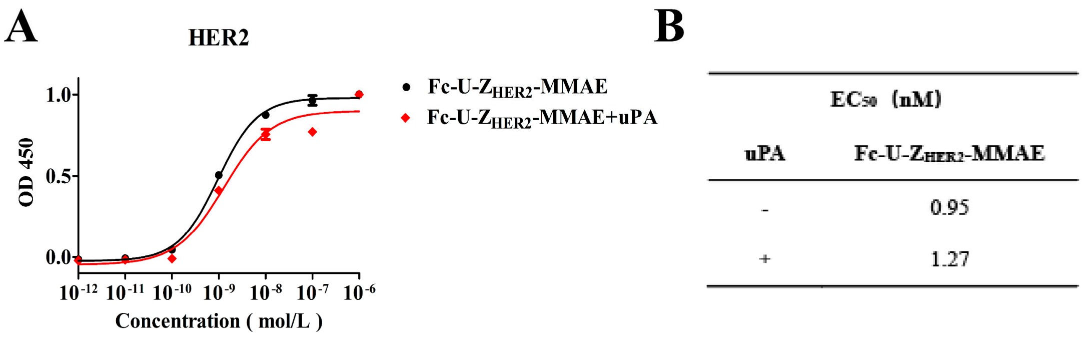

B

EC50 (nM)	
uPA	Fc-U-ZHER2-MMAE
-	0.95
+	1.27

Fig. S5. Binding ability of Fc-U-Z_{HER2}-MMAE after uPA cleavage. (**A**) ELISA of Fc-U-Z_{HER2}-MMAE after uPA cleavage. Fc-U-Z_{HER2}-MMAE was incubated with 400 nM uPA at 37 °C for 2 h and then analyzed with mouse anti-MMAE monoclonal antibody by ELISA against a tag-free HER2 protein. Data are expressed as the mean ± SEM (n = 3). (**B**) EC_{50} data calculated with GraphPad Prism 5.0 software.

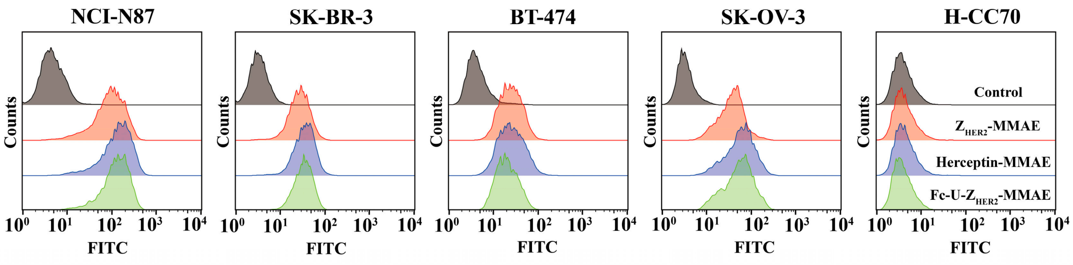

Fig. S6. Flow cytometry analysis of the binding specificity of the conjugates with cells with different levels of HER2 expression. The HER2-overexpressing cell lines NCI-N87, SK-BR-3, BT-474, and SK-OV-3 and the HER2-negative cell line H-CC70 were incubated with 10 nM conjugate for 2 h and then with a mouse anti-MMAE monoclonal antibody for 1 h. After incubation with a goat-anti-mouse IgG–FITC conjugate antibody, the cells were collected and analyzed by flow cytometry.

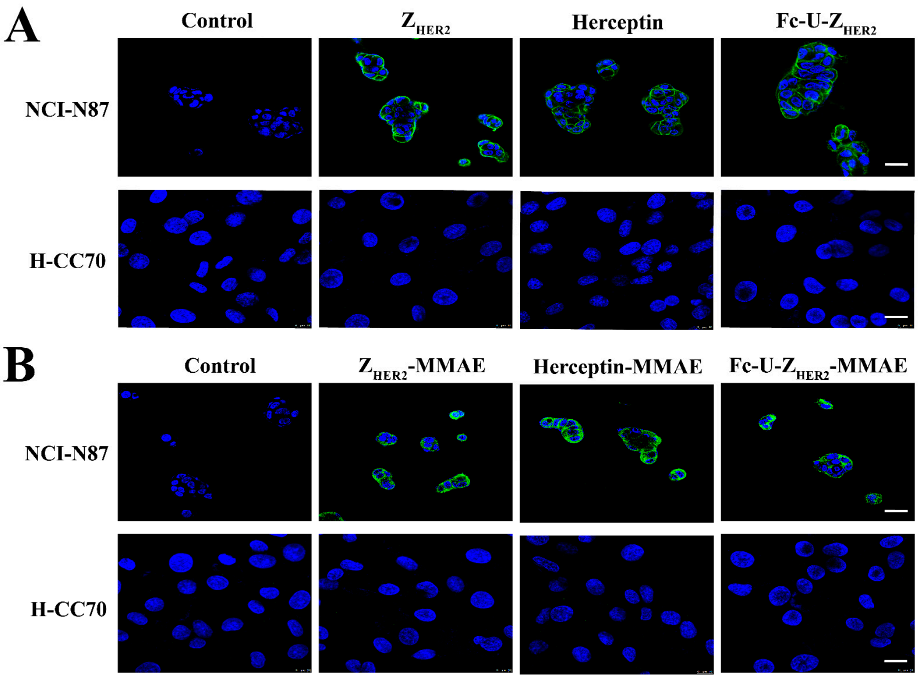

Fig. S7. Binding specificity of the conjugates and raw proteins visualized by confocal imaging. **(A&B)** Confocal imaging of the cells after incubation with the conjugates and raw proteins. NCI-N87 and H-CC70 cells were incubated with 100 nM protein without MMAE conjugated (**A**) or the conjugate (**B**) at 37 °C for 2 h, followed by mouse anti-His monoclonal antibody or mouse anti-MMAE monoclonal antibody for 1 hour and a goat-anti-mouse IgG-FITC

conjugate antibody for 1 hour. Observation was performed with a laser scanning confocal microscope. Scale bar for NCI-N87 cells: 25 μm; scale bar for H-CC70 cells: 15 μm.

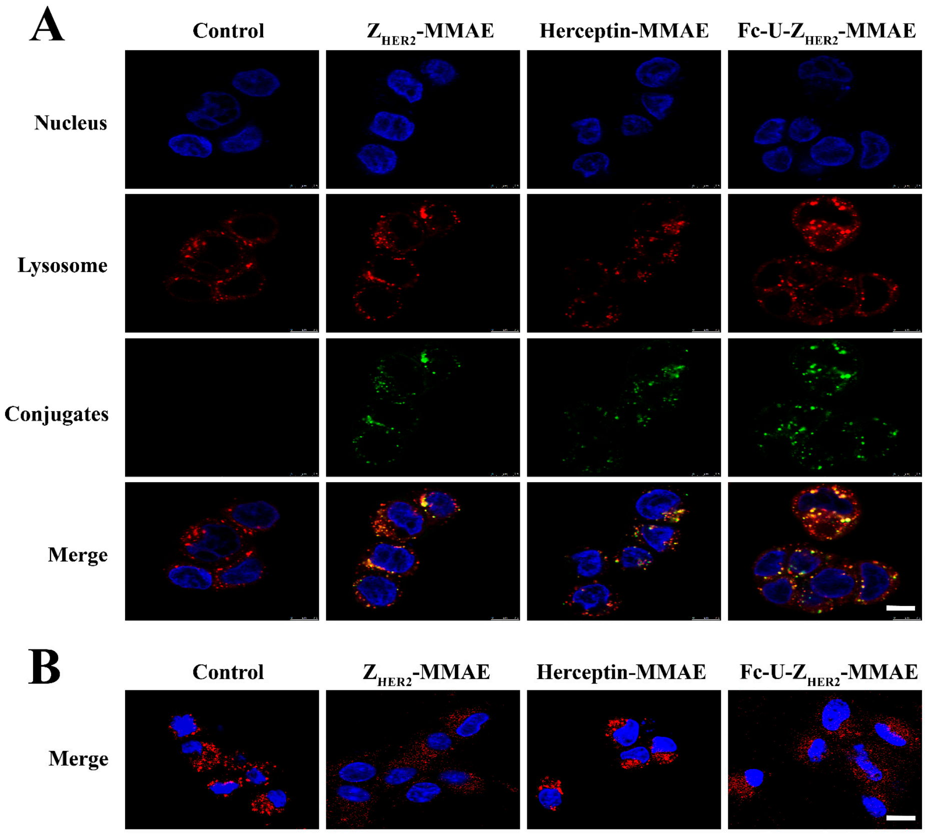

Fig. S8. Colocalization of the conjugates with lysosomes observed by CLSM. (**A&B**) CLSM imaging. HER2-overexpressing NCI-N87 cells (**A**) and HER2-negative H-CC70 cells (**B**) were incubated with 100 nM FITC-labeled conjugates at 37 °C for 4 h, followed by staining with Hoechst 33342 (nuclei, blue) and LysoTracker Red DND-99 (lysosome, red) and observation by CLSM. Scale bars: 7.5 μm.

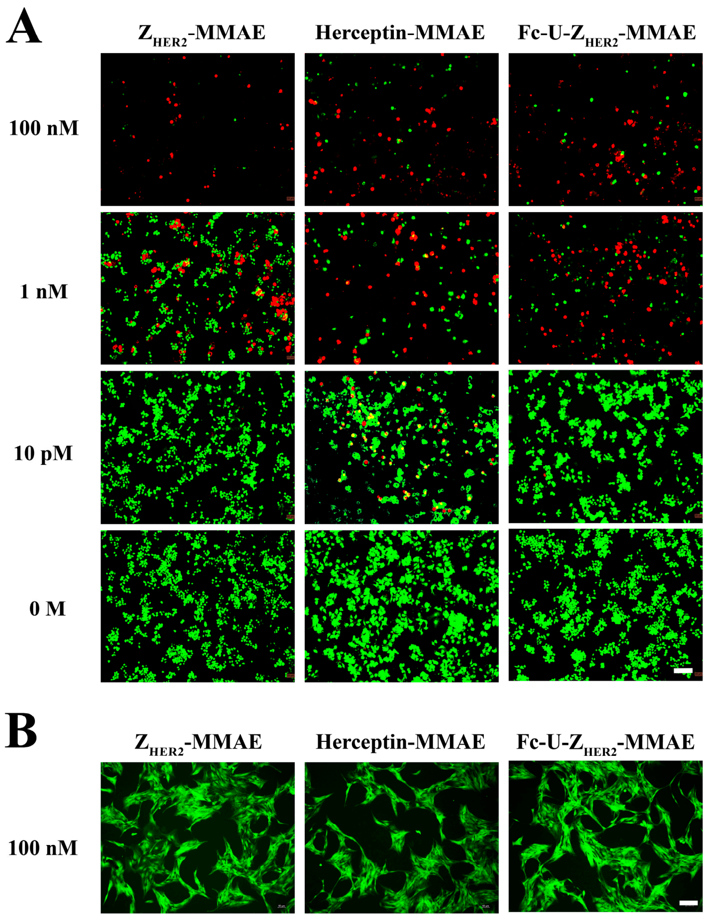

Fig. S9. Live/dead staining images for detection of the cytotoxicity of the conjugates. (**A&B**) CLSM imaging of the Live/dead staining. HER2-high expression SK-BR-3 cells (**A**) were incubated with 0, 0.01, 1 and 100 nM conjugates for 72 h, or HER2-low H-CC70 cells (**B**) were incubated with 100 nM conjugates for 72 h, followed by staining with Calcein AM (green, live) and EthD-1 (red, dead), and then visualized by fluorescence microscopy imaging. Scale bars: 100 μm.

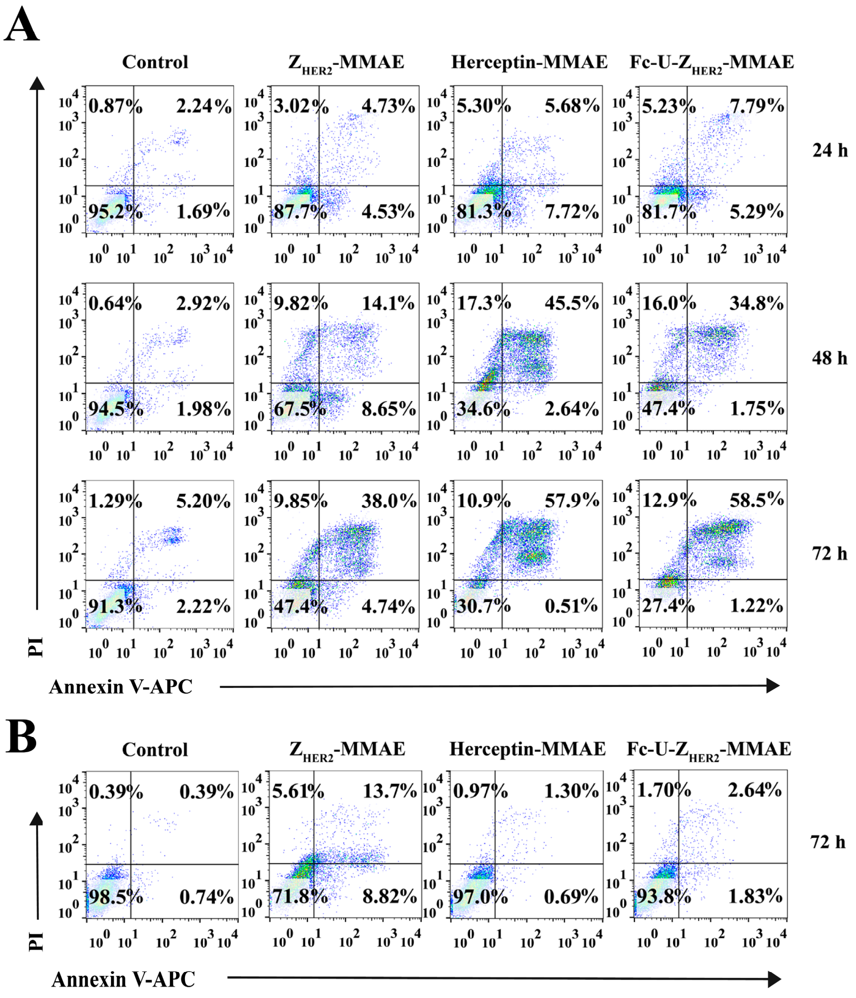

Fig. S10. FACS analysis of the apoptosis mechanism induced by the conjugates. HER2-overexpressing SK-BR-3 cells (**A**) were incubated with 10 nM conjugates for 24, 48, and 72 h, or HER2-negative H-CC70 cells (**B**) were incubated with 10 nM conjugates for 72 h, stained with Annexin V-APC/PI and analyzed by flow cytometry.

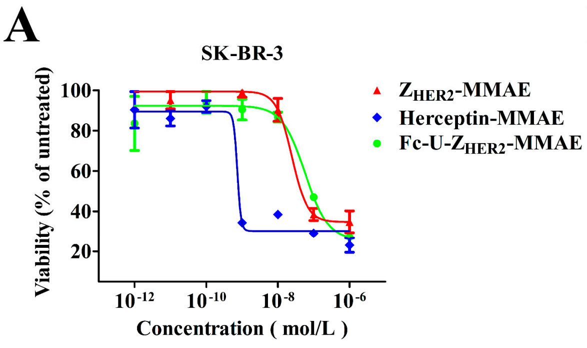

B

IC50 (nM)	
Conjugates	SK-BR-3
Z_{HER2}-MMAE	24.7
Herceptin-MMAE	0.738
Fc-U-Z_{HER2}-MMAE	57.3

Fig. S11. Competitive MTT assay to determine cell viability after receptor blockade. (**A**) Viability of SK-BR-3 cells after receptor blockade. The cells were preincubated with 5 μM Z_{HER2} for 1 h before the conjugates were added, and then the cell viabilities were determined

by MTT assays. Data are expressed as the mean ± SEM (n = 3). (**B**) IC_{50} data calculated with GraphPad Prism 5.0 software.

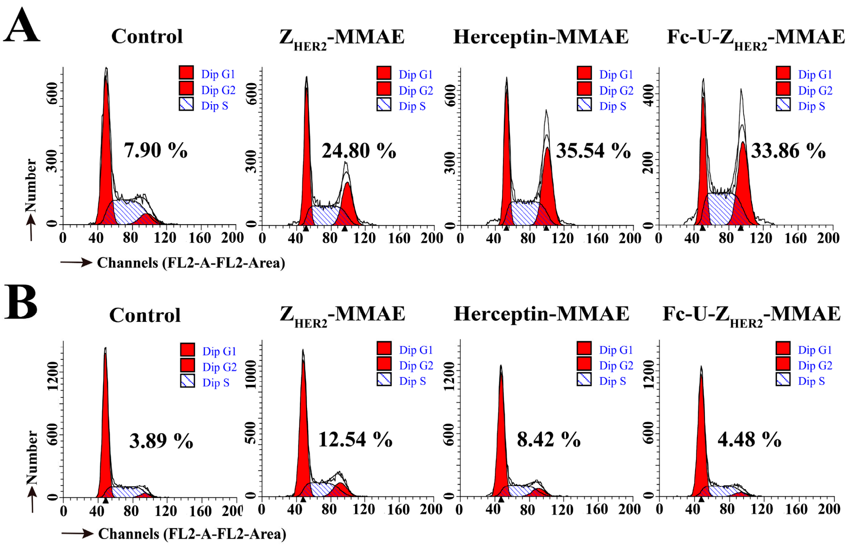

Fig. S12. Selective induction of G2 growth arrest by the conjugates. (**A&B**) Flow cytometry analysis of the cells after treatment with the conjugates. HER2 highly expressing NCI-N87 cells (**A**) and HER2-negative H-CC70 cells (**B**) were incubated with 100 nM conjugates for 18 h and then stained with PI before analysis by flow cytometry. The percentage of cells arrested at the G2/M phase is expressed as % G2/M.

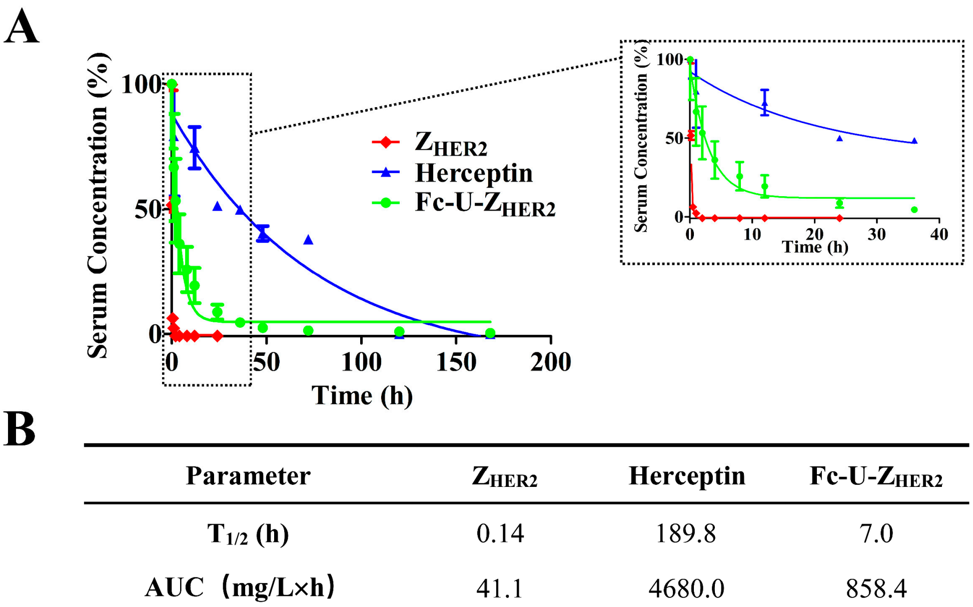

B

Parameter	Z_{HER2}	Herceptin	Fc-U-Z_{HER2}
$T_{1/2}$ (h)	0.14	189.8	7.0
AUC (mg/L×h)	41.1	4680.0	858.4

Fig. S13. Pharmacokinetics of Herceptin, Z_{HER2} and Fc-U-Z_{HER2} in mice. (**A**) Serum concentrations of the proteins without MMAE conjugation. The raw proteins were i.v. injected into BALB/c mice, and their concentrations in serum at different time points (n = 3) were quantified by ELISA. (**B**) Values of $t_{1/2}$ and AUC calculated with GraphPad Prism 5.0 software.

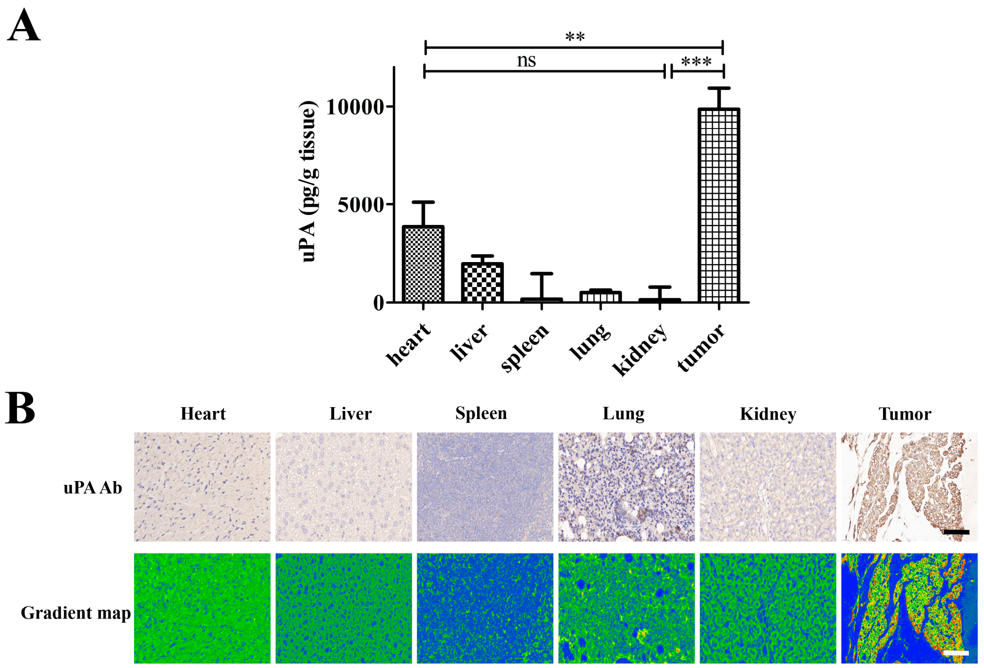

Fig. S14. Detection of uPA enzyme expression in various tissues of tumor-bearing mice by ELISA and immunohistochemistry. (**A&B**) ELISA and immunohistochemical analysis of the concentration of the uPA enzyme in organs and tumors. Organs and tumor tissues of NCI-N87-bearing mice were collected, and uPA enzyme expression was determined by a uPA enzyme detection kit after homogenization (**A**) or by immunohistochemical analysis (**B**). The gradient map was used to more intuitively display the positive signals of the immunohistochemical results, where blue was the background color, green was the cells and cytoplasm, and orange was the positive signal of uPA enzyme. Scale bars: 50 μm. Data represent the mean ± SEM (n = 3). Statistical significance was determined by one-way ANOVA with Tukey's multiple comparisons with GraphPad Prism 5.0 software. * $p < 0.05$, ** $p < 0.01$, *** $p < 0.001$, **** $p < 0.0001$.

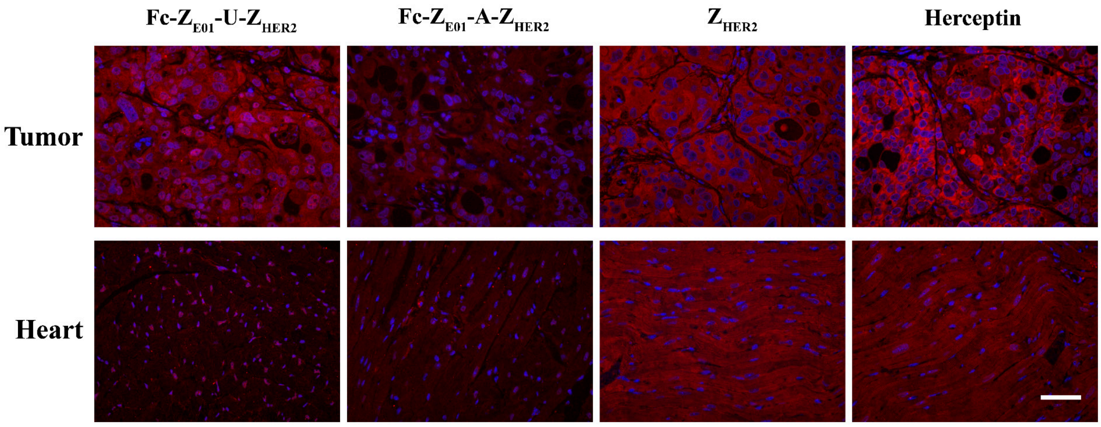

Fig. S15. Detection of HER2 receptor expression and proteolytic activity of uPA enzyme in

heart and tumor tissue from NCI-N87 tumor-bearing mice. Frozen tumor and heart sections were incubated with Fc-Z_{E01}-U-Z_{HER2}, Fc-Z_{E01}-A-Z_{HER2}, Z_{HER2}, or Herceptin and then incubated with anti-His antibodies, followed by staining with Cy3-labeled goat anti-rabbit IgG antibody (red) and DAPI (blue). Scale bars: 50 μm.

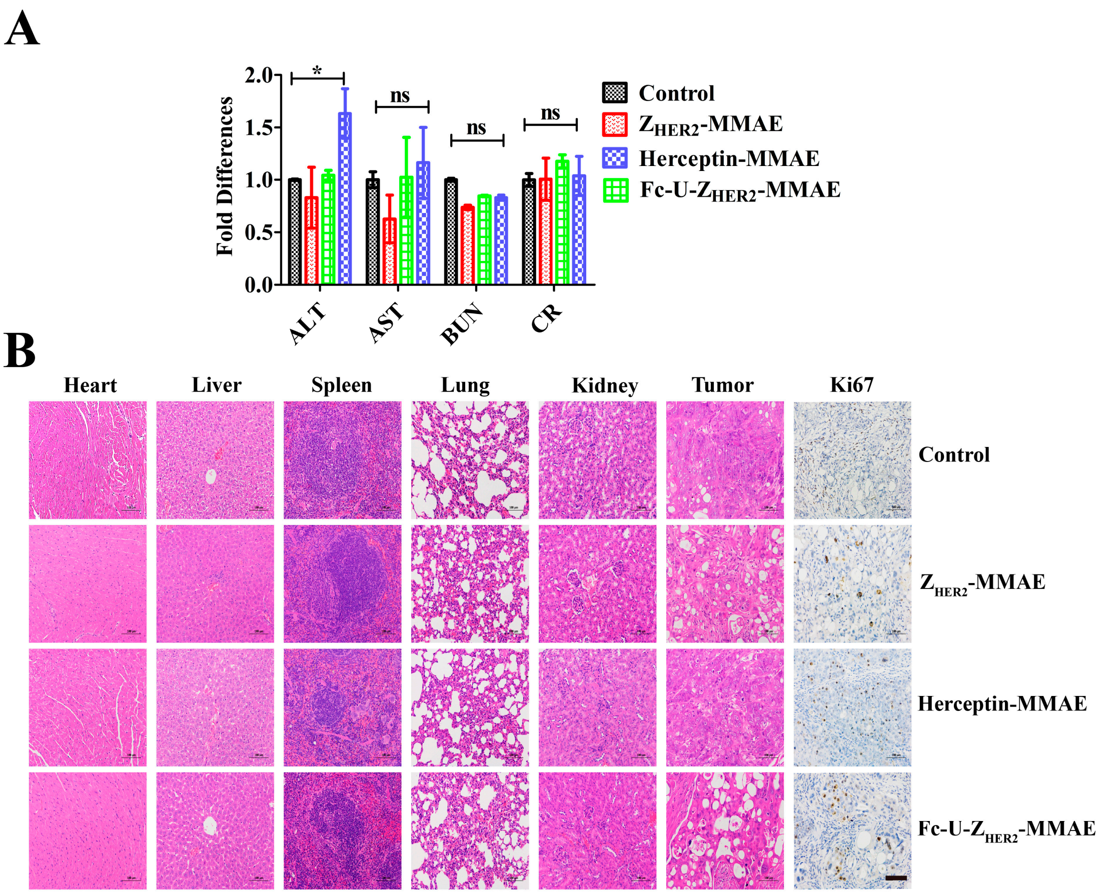

Fig. S16. Biochemical analysis of the serum and H&E staining of the mouse organs after treatment with six doses of the conjugates. (**A**) Serum levels of ALT, AST, BUN, and CR in the mice after treatment. Blood samples were collected, and serum was separated to measure the ALT, AST, BUN, and CR levels. Error bars, means ± SEMs (n = 3). Statistical significance was determined by one-way ANOVA with Tukey's multiple comparisons with GraphPad Prism 5.0 software. * $p < 0.05$, ** $p < 0.01$, *** $p < 0.001$; **** $p < 0.0001$; ns = not significant. (**B**) H&E and Ki-67 staining of the organs and tumors from the mice after treatment. Scale bars: 100 μm.
